# Teaching Strategies and Psychological Effects of Entrepreneurship Education for College Students Majoring in Social Security Law Based on Deep Learning and Artificial Intelligence

**DOI:** 10.3389/fpsyg.2022.779669

**Published:** 2022-03-17

**Authors:** Qinlei Zhu, Hao Zhang

**Affiliations:** Law School, Shanghai University of Finance and Economics, Shanghai, China

**Keywords:** deep learning, social security law, active personality, entrepreneurial education, entrepreneurial ability and entrepreneurial intention

## Abstract

This study aims to achieve the goal of cultivating and reserving emerging professional talents in social security law, improve the curriculum and mechanism of entrepreneurship education, and improve students’ entrepreneurial willingness and entrepreneurial ability. Deep learning technology is used to study the psychological effects of entrepreneurship education for college students majoring in social security law. Firstly, the concept of entrepreneurial psychology is elaborated and summarized. A related model is designed using the theory of proactive personality and planned behavior through questionnaire survey and regression analysis to explore the relationship between students’ entrepreneurial psychology and entrepreneurial intention. Secondly, an entrepreneurship education method based on deep learning is proposed, and a teaching model of multi-dimensional collaborative entrepreneurship education practice is constructed. On this basis, the deep learning algorithm combines the characteristics of the personalized recommendation algorithm to construct an efficient Problem-Based Learning (PBL) learning resource recommendation algorithm. Finally, the proposed method is tested. The results show that the Significant (Sig.) value of students who have participated in PBL deep learning courses is less than 0.05, indicating that PBL significantly improves students’ learning ability and the ability to deal with entrepreneurial environments. The results verify the impact of entrepreneurial learning on entrepreneurial intentions. The research on PBL online learning recommendation system shows that the proposed recommendation algorithm is superior to the traditional recommendation algorithm in both roots mean square error value and mean absolute error value on both datasets. The proposed method provides a new idea of reform and innovation to cultivate social security law professionals and the cultivation of the reserve model.

## Introduction

With the advent of globalization and the era of “Internet+” information intelligence, education is also facing continuous reform and innovation challenges. The disadvantages of traditional teaching modes such as “teacher centrism and duck-stuffing teaching” are gradually recognized and discovered by people. While Problem-Based Learning (PBL) teaching conforms to the needs of the development of the times, subverts the traditional teaching model, and focuses on learners’ autonomous learning ability (Bernal et al., 2019). Teachers guide students to explore in problem situations actively and jointly seek problem solutions in group cooperation, thus improving learners’ classroom participation and enhancing students’ awareness of problems, innovation, and collaboration. Although the specific expressions are different, the teaching concept of PBL teaching and deep learning has changed from “teaching is transmission” to “teaching is dialogue,” both pointing to students’ independent inquiry under the guidance of teachers (Stefanis, 2020). Because of this, PBL teaching is regarded as an effective way to realize further teaching. In the open PBL teaching course, teachers use real and challenging problems to drive students’ thinking, and promote students to absorb curriculum knowledge firmly (Bur et al., 2021), deeply study the essence of geography, and form the learning framework and thinking mode of geography through group cooperation and problem-solving, to achieve the state of deep learning (Huang, 2021). In terms of teaching practice, researchers have found the impact of deep learning on students’ critical thinking and problem-solving ability, and widely applied deep learning to many fields such as medicine and pedagogy (Yuan et al., 2020).

Zhu et al. (2019) proposed that deep learning is the revelation and deep understanding of the essence of effective learning, which aims to improve people’s advanced thinking ability and problem-solving ability. The teaching logic of deep learning consists of four elements: students’ cognitive order, subject content characteristics, subject core competence, and learning effect reflection. Deep learning is one of the important research topics in educational technology that emphasizes higher cognitive goals and higher thinking ability (Zu, 2019), to realize learners’ further learning, introduced the teaching mode of Small Private Online Course (SPOC), and analyzed its advantages and characteristics. He also expounded the important role in developing deep learning motivation, deep learning participation, deep learning strategies, and deep learning results. Exploring the SPOC-based teaching mode can realize the deep integration of information technology and education, promote further learning, and improve the learning effect. In the intelligent classroom environment, students’ emotional content analysis plays a vital role because it helps cultivate an emotional state conducive to learning (Gupta et al., 2019) proposed a student emotional content analysis method based on the maximum edge face detection. The emotional content analysis includes the analysis of students’ four different emotions, namely: high positive emotion, low positive emotion, high negative emotion, and low negative emotion. The participation score is calculated according to the four emotions predicted by the proposed method. In addition, the whole classroom is regarded as a group, and the corresponding group participation score is used to analyze the classroom participation. The emotional content videos fed back and analyzed by experts are used as feedback to teachers to improve teaching strategies and students’ learning rate. In the era of big data, artificial intelligence (AI) has been widely used in higher education, providing technical support for practical teaching in colleges and universities (Yang et al., 2020) created a practical teaching model based on AI. An intelligent management cloud platform is constructed for practical teaching based on the advantages of massive open online courses (MOOC) and SPOC. Meanwhile, AI technology is used to realize personalized learning and provide intelligent push services. In this way, the online MOOC + SPOC platform seamlessly integrates the teaching content into specific teaching scenarios, and the offline cloud platform manages the teaching process intelligently. Under the proposed teaching mode, the teaching content can match the working ability standard and the actual ability of college students. With the popularization of AI and the continuous development of information teaching, the emergence of smart classrooms provides a new teaching environment for classroom teaching. As a new and efficient teaching mode, a smart classroom has entered the school (Yang et al., 2019), based on the classical PBL teaching method, built a problem-driven combinatorial mathematics smart classroom and shared some teaching experience from the aspects of teaching preparation, teaching implementation, and teaching evaluation. Additionally, the integrated development of AI and the financial industry has accelerated the rise of financial technology, put forward higher requirements for the training of financial engineering professionals and the teaching of “financial data mining” (Guan et al., 2021) summarized the teaching experience of the course and proposed the reform of the PBL teaching mode of financial data mining. Moreover, they carried out reform and exploration regarding course objectives, teaching contents, teaching methods, course evaluation, and problem-solving strategies. The relevant literature revealed that deep learning and PBL research started relatively late. In terms of deep learning, foreign research mainly focuses on theoretical analysis, learning environment, and application field. The studies on deep learning in China have also been widely concerned by scholars in many fields and have achieved some research results, but there are still deficiencies in the research depth and breadth. First, the research depth of theoretical basis and learning environment is not enough. Second, the existing studies fail to combine theory with practice or apply to practical teaching effectively, and the application breadth is not enough. In the aspect of PBL, related theoretical research has laid the research foundation; regarding the evaluation research, PBL-based teaching mode focuses on students’ process and developmental evaluation, and its evaluation method is more scientific and reasonable; in terms of application and promotion, foreign research is relatively mature, and PBL-based teaching mode has been widely used in many disciplines such as medicine and basic education. China is still in the primary stage of development compared with other countries. The wide application of digital teaching in China’s basic education has become the most important teaching auxiliary means. However, the PBL teaching mode in the digital learning environment has not been popularized on a large scale, which is inseparable from the actual conditions in China. On the one hand, primary and secondary school students have poor self-control, and learners are very vulnerable to other network information interference when using network equipment. On the other hand, the intelligent learning environment based on terminals and e-book packages is affected by regional and economic factors, which hinders its development process to a great extent. Based on this, this teaching design starts from the traditional classroom. It takes the existing teaching media as an auxiliary to carry out PBL teaching for further learning, opens up a new path for stimulating students’ autonomous learning and innovation ability, and promotes students’ further learning.

Innovation and entrepreneurship teaching in colleges and universities is an important model for the construction and development of modern colleges and universities, and it is also the main direction for the cultivation of new talents in modern society. The survey shows that the proportion of innovation and entrepreneurship among college graduates in developed countries is between 20 and 30%. The proportion of innovation and entrepreneurship in China is less than 10%. In terms of the number of innovation and entrepreneurship, the number of innovation and entrepreneurship in developed countries is 2–3 times that of China. At present, the teaching of innovation and entrepreneurship in Chinese colleges and universities plays a vital role in the cultivation of talents and the progress of new technologies. The development of innovation and entrepreneurship teaching is one of the important ways for the development of AI technology. The efficient promotion of China’s innovation and entrepreneurship teaching model is conducive to the integration of innovation and entrepreneurship teaching and AI technology. The integration of AI technology and innovation and entrepreneurship education enables Chinese colleges and universities to complete the reform of innovation and entrepreneurship education mode, and to maximize the level of innovation and entrepreneurship education in colleges and universities. The integration of artificial intelligence technology research and training with innovation and entrepreneurship education in colleges and universities can promote the common progress of the two to a certain level and achieve the purpose of better serving the society.

The innovation points can be divided into two points: 1. implement the entrepreneurial model with dynamic personality and planned behavior as the mediating effect, and analyze the performance of the model through questionnaires. 2. The PBL teaching model for deep learning is divided into three stages: problem creation, problem solving, evaluation and reflection. The contribution lies in the research on the mediating factors in entrepreneurial psychology and the establishment of the entrepreneurial education model based on the PBL method. These include the determination and guidance of entrepreneurial goals, the optimization of teaching materials, the stimulation of interest and potential, and the practical interaction between teachers and students. The purpose is to promote the innovation of the talent training model of the social security law major and adapt to the new era of increasingly severe employment pressure.

Research methods are divided into three types:

(1) Literature analysis method

The literature on PBL teaching and deep learning is studied. The concepts and characteristics of deep learning and PBL learning are organized. The representation of deep learning in PBL is analyzed. The PBL teaching model is constructed from the perspective of deep learning.

(2) Questionnaire survey method

The six dimensions of innovation, control, endurance, risk-taking, self-confidence, and achievement are designed by questionnaire. There are 25 questions in the questionnaire. The purpose is to understand the students’ entrepreneurial intention.

(3) Experimental method

The experimental method is used to test the performance of the constructed PBL online learning recommendation system.

## Construction of Deep Learning Problem-Based Learning Method for Entrepreneurial Psychology

### Entrepreneurship Learning and Entrepreneurial Intention

#### Entrepreneurship Learning

In 1998, entrepreneurial learning was proposed as an independent concept, but it was not uniformly defined. Entrepreneurship learning expresses the learning behavior of entrepreneurs who accumulate the resources and abilities required for entrepreneurship by reflecting on and summarizing past experiences and observing the behavior of others (Rogoza et al., 2018; Macneil and Schoonmaker, 2020). Entrepreneurship learning is the deep processing of experience and cognition by entrepreneurs and reconstructing the knowledge and skills they originally possessed according to the needs of entrepreneurial activities (Funken and Gielnik, 2020). In the face of uncertainty, entrepreneurs can find the optimal solution to the problems encountered in entrepreneurial activities through entrepreneurial learning, and entrepreneurial learning runs through the entire entrepreneurial activity. Generally, the process of entrepreneurial learning is divided into three levels, namely experiential learning, cognitive learning, and practical learning (Teymurova et al., 2020). Its influencing factors generally include personal emotions, social networks, and failure experience. Entrepreneurship behavior typically impacts entrepreneurs’ abilities, intentions, and cognition.

#### Entrepreneurship Intention

Entrepreneurship intention refers to the individual’s intentions and ideas for entrepreneurial activities. It determines whether the individual has the behavioral tendency and possibility to choose entrepreneurial activities (Annisa et al., 2021). Entrepreneurship intention is a mental state of entrepreneurs focusing their energy, experience, resources, and attention on entrepreneurial behavior. The entrepreneurial intention has been generated before entrepreneurial behavior occurs, and it is the best predictive criterion for judging entrepreneurial behavior (Wu and Song, 2019). Generally, the factors that affect entrepreneurial intentions will be divided into personal characteristics and social aspects.

Personality is often regarded as a criterion that can predict whether an individual will engage in entrepreneurial behavior among the personal factors. Personality represents whether an individual can actively and actively change himself. Individuals who become entrepreneurs and not entrepreneurs have significant personality differences. Among the environmental factors, when individuals are in an environment with a robust entrepreneurial culture, they will think that entrepreneurial behavior is more rationalized, which will result in stronger entrepreneurial intentions. Such an environment is usually reflected in the individual with a high sense of self-efficacy, social network, and the entrepreneurial education courses received in his school (Wu et al., 2019).

### Modeling the Mesomeric Effect of Active Personality and Theory of Planned Behavior

#### Active Personality

Active and passive individuals with positive characteristics can often quickly identify and grasp opportunities that are beneficial to development. Facing changes in the entrepreneurial environment, they usually have the ability, and intention to actively change the background or even create the experience. Passive individuals with negative characteristics often take an indifferent attitude to the environment and its changes (Tarabashkina et al., 2020). The vibrant personality is usually less constrained by the environment. It is good at tapping the favorable opportunities in the background to take measures and solve problems with a positive attitude. According to the concept of dynamic personality, the research defines it as a stable individual who has positive characteristics looking for new opportunities and ways and actively takes measures to change the environment and the pressure brought about by it.

#### Modeling the Theory of Planned Behavior

**FIGURE 1:**
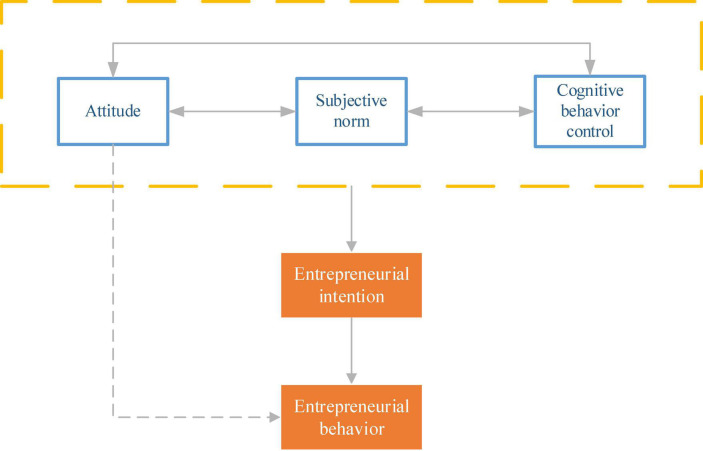
Planning behavior relationship modeling.

Theory of Planned Behavior extends a rational behavior theory proposed in 1991 (Japutra and Molinillo, 2019; Zhou, 2019; Abdollahzadeh and Amin, 2020). The Theory of Planned Behavior believes that the three influencing factors of entrepreneurial intention are attitude, subjective norms, and cognitive behavior control. These elements act on entrepreneurial intention, promoting entrepreneurial behavior. Among these three elements, an attitude refers to an individual’s perception and evaluation of a particular behavioral activity, reflecting the possibility of an individual taking behavior. Subjective norms refer to: when individuals are affected by the external environment, they make certain behavioral decisions; cognitive behavior control refers to the judgments made by individuals as to whether they can achieve their goals when performing certain activities (Graham and Rosén, 2019). These three elements are the root cause of an individual’s entrepreneurial intention and positively correlate with the individual’s entrepreneurial sense. The relationship between entrepreneurial intention and entrepreneurial behavior is modeled, as shown in Figure 1.

In Figure 1, entrepreneurial intentions directly impact entrepreneurial behavior under ideal circumstances. However, this relationship is often affected and restricted by factors such as the individual’s ability, the opportunity encountered, the recognition of the opportunity, and the resource conditions owned by the individual. In the Theory of Planned Behavior relationship, absolute cognitive behavior control can accurately predict the possibility of entrepreneurial behavior (Neuberger and Pabian, 2019). Attitudes, subjective norms, and cognitive behavior control are three variables that act on entrepreneurial intentions, independent of but related to each other. The theoretical model of planned behavior includes 1. Under ideal conditions, entrepreneurial intention can directly impact entrepreneurial behavior. But it will be affected by conditions such as personal ability, opportunity identification, and resources. 2. True cognitive-behavioral control can accurately predict the likelihood of behavior occurring. 3. Attitude, subjective norm, and cognitive-behavioral control are the three main variables that affect intention, and the three act together on intention to promote behavior. 4. The three variables are not only independent of each other but also related to each other.

#### Effect Relationship Modeling

Entrepreneurship learning is a behavior that runs through the entire process of entrepreneurial behavior. The Theory of Planned Behavior provides a theoretical framework for its relevance to entrepreneurial intentions. After learning through entrepreneurship, individual students’ attitudes and cognitive behavior control may change, affecting their entrepreneurial intentions, thereby promoting entrepreneurial behavior. The judgment made by potential entrepreneurs toward the external environment is the attitude in the Theory of Planned Behavior. In entrepreneurial learning, potential entrepreneurs can obtain more entrepreneurial information and entrepreneurial experience through learning and reflection. After the possible entrepreneurial individuals transform and absorb the entrepreneurial knowledge they have learned, they can improve their entrepreneurial skills and confidence, thus positively impacting their attitudes. Evaluating potential entrepreneurial individuals in entrepreneurial activities is the control of cognitive behavior. When entrepreneurial individuals are placed in an environment with a robust entrepreneurial atmosphere, they can enrich their cognition through learning and practice, promoting the generation and improvement of entrepreneurial intentions. In summary, the hypothesis is obtained:

T1: Entrepreneurship learning and entrepreneurial intention are positively correlated;
T1a: Experience learning in entrepreneurial learning is positively correlated with entrepreneurial intentions;
T1b: Cognitive learning in entrepreneurial learning is positively correlated with entrepreneurial intention;
T1c: Practical learning in entrepreneurial learning is positively correlated with entrepreneurial intentions.

According to research on dynamic personality in enterprises, vivacious personality can play a positive moderating role in sharing information, knowledge, self-esteem, employee empowerment, and negative regulation between the distribution of power and the perception of employee empowerment. Facing the external environment, individuals with dynamic personalities can respond quickly and adjust and change, which has a positive correlation with entrepreneurial learning. Enthusiastic personality can directly or indirectly affect entrepreneurial intentions, and this indirect effect is accomplished through healthy competition. When potential entrepreneurs have a healthy competitive attitude and atmosphere, their entrepreneurial intentions will be stronger. The proposed method believes that individuals with dynamic personalities are rarely or even unconstrained by the environment, can actively respond to, change, and even create opportunities and backgrounds, and have a positive attitude toward learning new things. Meanwhile, individuals with dynamic personalities can take the initiative to take measures against the pressures and problems of the entrepreneurial environment and make entrepreneurial choices. Therefore, dynamic character plays a positive intermediary role in strengthening the relationship between entrepreneurial learning and entrepreneurial intention. In other words, whether possessing a vibrant personality will affect entrepreneurial education on entrepreneurial intentions. In contrast, individuals possessing a dynamic nature will have a more substantial effect. In summary, the hypothesis is obtained:

T2: Active personality plays a moderating role between entrepreneurial learning and entrepreneurial intention;
T2a: Active personality plays a moderating role between experiential learning and entrepreneurial intention;
T2b: Active personality plays a moderating role between the cognitive learning and entrepreneurial intention;
T2c: Active personality plays a moderating role between practical learning and entrepreneurial intentions.

**FIGURE 2:**
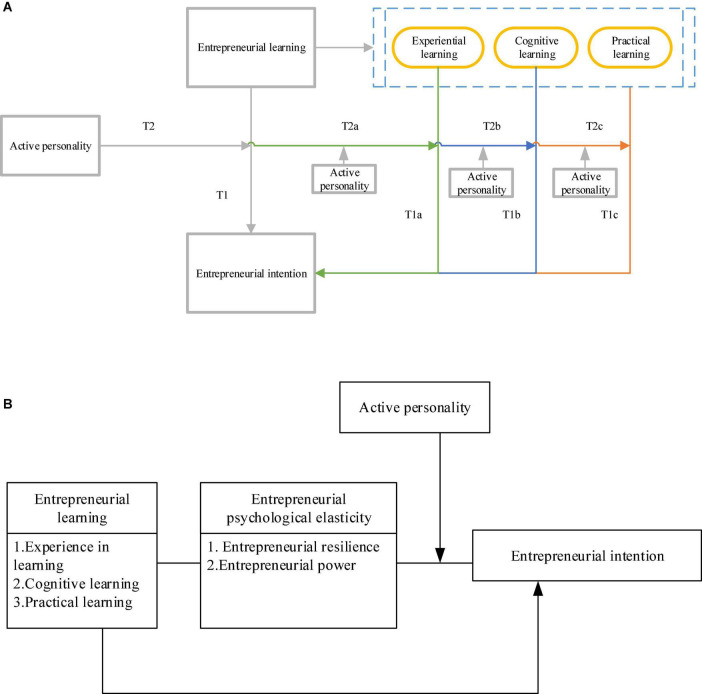
Relational model. **(A)** Influence relationship modeling **(B)** Research model.

The proposed method defines entrepreneurial learning as an independent variable, dynamic personality as a moderating variable, and entrepreneurial intention as a dependent variable. The proposed method analyzes the relationship between the three. The assumptions obtained are modeled, and the relationship modeling is shown in Figure 2.

## Research Model

### Problem-Based Learning Deep Learning Method

#### Deep Learning

In the field of education, deep learning content tends to be learners’ motivation, knowledge experience, situation, thinking level, and ability level. It requires learners to use a variety of channels to obtain information and analyze and integrate the data. Meaning is actively constructed. Unique knowledge linkages are built. Thereby, the transfer of knowledge and its application in practical situations are realized, and the process and results of problem-solving are reflected and judged (Wu J. et al., 2020; Yuan and Wu, 2020). Deep learning in education is a kind of learning-oriented to higher-order thinking activities, with comprehensive learning of initiative and practice, understanding and criticism, reflection, and innovation.

In AI (AI), deep learning is a new field in machine learning research. The motivation is to build a neural network that simulates the human brain for analysis and learning, which mimics the mechanism of the human brain to interpret data such as images, sounds, and texts. Deep learning is a type of unsupervised learning. The deep learning algorithm used in the constructed PBL online learning recommendation system is Auto Encoder, a neural network whose input is approximately equal to the output. As an unsupervised model, the Auto Encoder can extract core features from the dataset and increase the expressiveness of the model. It can also increase the number of layers and hidden neurons in the neural network. When performing feature representation or feature extraction, the sparsity of features can better extract independent and high-quality components. Therefore, scholars proposed Sparse Auto Encoder (SAE). SAE adds a sparse before the traditional Auto Encoder so that the representation of each neuron satisfies a certain sparsity. It is expressed by adding a sparse prior constraint to the loss function. $a_{j}^{\left(2\right)}$ represents the activation of the hidden neuron *j* in the second layer. $a_{f}^{\left(2\right)}\,\left(x\right)$ represents the activation degree (that is, the output value) of the hidden neuron *j* of the autoencoder neural network when the input is *x*. The average activation of neuron *j* can be defined as Equation (1):


(1) $$\dot{\hat{\rho }}=\frac{1}{m}\,\sum_{i=1}^{m}\left[a_{j}^{\left(2\right)}\,\left(x^{\left(i\right)}\right)\right]$$

The sparsity parameter ρ is introduced. ρ is defined as a number close to 0 such that ${\hat{\rho }}_{j}=\rho$. In this way, the neuron’s output can be made as 0 as possible to achieve sparsity. An additional penalty factor is added to the optimization objective function to achieve this constraint. In the SAE model, the relative entropy (KL divergence) is used as a penalty factor, as shown in Equation (2):


(2) $$\sum_{j}K\,L\,\left(\rho ∥{\hat{\rho }}_{j}\right)=\sum_{j}\left[\rho \,\text{log}\,\frac{\rho }{{\hat{\rho }}_{j}}+\left(1-\rho \right)\,\text{log}\,\frac{\left(1-\rho \right)}{\left(1-{\hat{\rho }}_{j}\right)}\right]$$

Finally, the overall cost function of SAE can be expressed as Equation (3):


(3) $$\begin{array}{c} J_{\text{sparre}}\,\left(W,b\right)=J\,\left(W,b\right)+\beta \,\sum_{j}K\,L\,\left(\rho ∥{\hat{\rho }}_{j}\right)\\ =\frac{1}{m}\,\sum_{l=1}^{m}\left(\frac{1}{2}\,{∥h_{W,b}\,\left(x^{\left(t\right)}\right)-x^{\left(i\right)}∥}_{2}^{2}\right)+\beta \,\sum_{j}K\,L\,\left(\rho ∥{\hat{\rho }}_{j}\right) \end{array}$$

#### Problem-Based Learning Method

**FIGURE 3:**
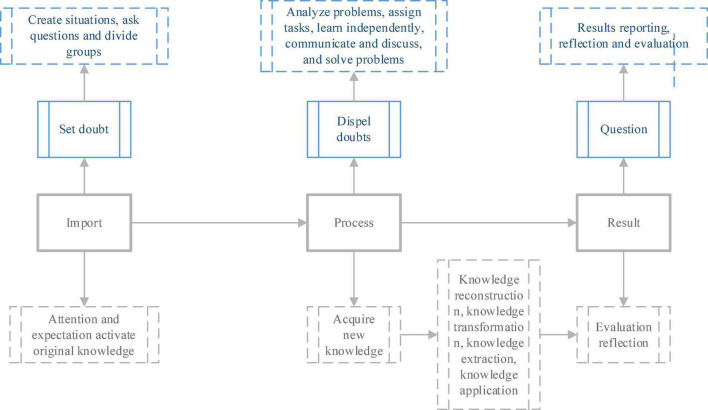
PBL method teaching process.

PBL method, a problem-based teaching model, focuses on the combination of theory and practice in education and emphasizes that the subject of education is students (Wu W. et al., 2020). In existing studies, the PBL teaching mode is generally understood as: a teaching method that allows students to collaborate in groups in a complex environment, use knowledge and skills to deal with and solve practical problems, and improve students’ independent learning and problem response and resolution ability (Wu et al., 2017). The proposed method defines the three elements of the PBL teaching model as lead-in and process result. It is defined as taking students as the subject and teachers as the problem setters. Students can decompose and solve the problems, build a knowledge framework and cooperation groups, and finally gain knowledge and skills. The learning process model is shown in Figure 3.

In the PBL teaching process shown in Figure 3, the lead-in phase can also be defined as the questioning phase. The instructor analyzes and diagnoses the content and goals of the teaching in combination with the characteristics of the students before class, and constructs the teaching content into a “problem” model (Zheng et al., 2018). In this way, the process of “problem” construction is a summary of knowledge and a multi-dimensional integration of knowledge to stimulate students’ curiosity and thirst for learning. The process stage is problem-solving: students’ study in groups, discuss problems with members, propose solutions, and summarize the knowledge and information to become a shared resource for the group. The resulting stage can also be defined as the questioning stage: under the instructor’s organization, students are divided into groups, and the groups collaborate to evaluate the learning and practical results to realize the training of reflection and judgment thinking.

#### Collaborative Filtering Algorithm

**FIGURE 4:**
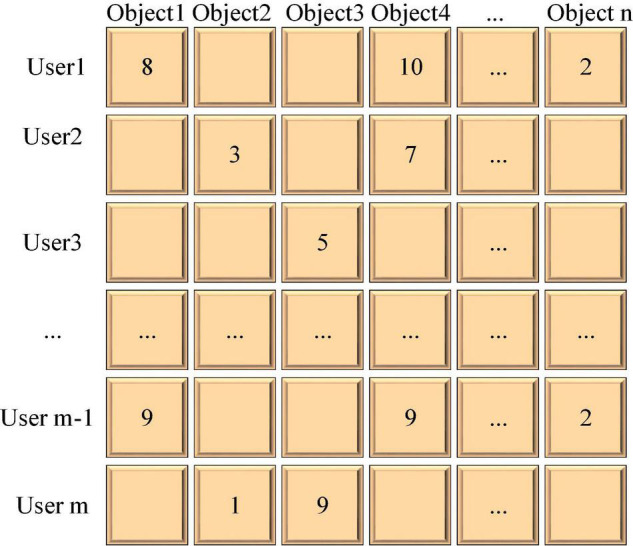
Evaluation matrix of user objects.

The Collaborative Filtering (CF) algorithm is currently the most practical and popular recommendation algorithm. The collaborative intelligence among the user groups is used to make recommendation judgments. Generally, there are two implementations of CF algorithm: the nearest neighbor-based CF algorithm and the model-based CF algorithm. The CF algorithm mainly uses the user’s historical behavior data to complete the recommendation. According to the different system designs, user behavior can be divided into explicit and implicit feedback. Explicit feedback refers to a module explicitly designed in the system to allow users to rate objects to express their preferences. User historical precise data are aggregated to form a User-Item evaluation matrix (m represents the number of users and n represents the number of objects). The user object evaluation matrix of CF algorithm is shown in Figure 4.

In Figure 4, each value in this matrix represents the user’s object rating. An empty position indicates that the user has not made a corresponding review. Implicit feedback means that the system does not have a corresponding scoring module contrasted with explicit feedback. The user’s preference is obtained by analyzing the user’s behavior. In general, behaviors can be converted into corresponding explicit scores, for example: browsing = 1 point, favorite = 2 points, download = 3 points, sharing = 4 points, etc. Therefore, the implicit rating data is transformed through the corresponding rules, and finally, the user object evaluation matrix is obtained.

#### Problem-Based Learning Online Learning Recommendation System

With the continuous deepening of “Internet + education,” the PBL online learning system has become a new way to acquire knowledge. However, there are problems such as homogeneity and insufficient personalization in the PBL online learning system. The current online learning system usually takes the teaching platform as the core. The learners take the pre-set learning resources in the platform as the primary learning content during the learning process. Still, there is a problem of homogeneity. When learners need personalized learning services, they need to find and select the required resources. This reduces the learning efficiency and learning experience to a certain extent. Therefore, how to improve the pertinence, personalization, and intelligence of teaching has become a challenge in the construction of the PBL online learning system.

The deep learning algorithm combined with the characteristics of the personalized recommendation algorithm is used to construct an efficient learning resource recommendation algorithm. It can effectively improve recommendation accuracy and enable learners to obtain a more personalized and intelligent learning experience when conducting PBL online learning. Since the performance in recommendation algorithms is susceptible to data sparsity conditions, the Supervised Neural Recommendation (SNR) model is proposed. The model uses the stacked autoencoder model to extract the learner’s learning behavior data, then uses its features for input restoration and scoring prediction to achieve the purpose of recommendation. In the model, the structured information of objects and users is used to construct the classification process, and the Huber function is used to constrain the model parameters. This effectively improves the accuracy of the recommendation model. The mainstream recommendation method types include CF algorithm, content-based recommendation, knowledge-based recommendation, and combination recommendation.

Since the Auto Encoder is proposed for image processing applications, it will inevitably face data sparsity when applied to the recommendation system application. In recommender systems, the input data is highly sparse. The User-Item matrix itself is exceptionally light. In a general recommender system, the sparsity of the User-Item matrix is 99%. Data sparsity can lead to the fact that the extracted features in the data may not be sufficient for data reconstruction or score prediction. Ninety-nine percent of the data is predicted with 1% of the data. The recommendation algorithm based on the Auto Encoder is extended, and other related data are introduced to solve the data sparsity and improve the recommendation effect.

**FIGURE 5:**
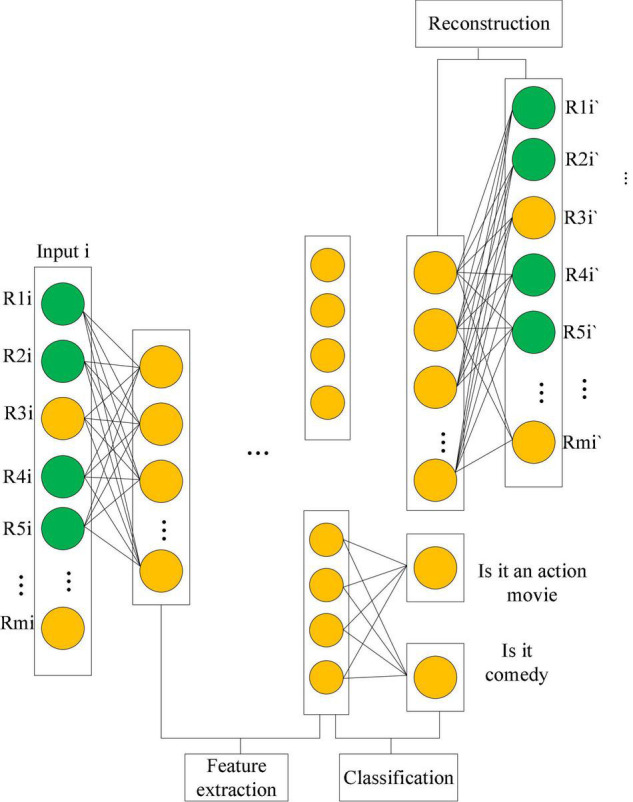
Example of recommendation model for SAE.

In general, in addition to the historical data of learners, online learning systems have structured features of objects or users in the system. These features can also be a good measure of its characteristics, such as the user’s age, occupation, gender, and other attributes. Release time, director, actors, etc., are influencing factors for movie recommendation. These structured data can help recommender systems build feature models of users and objects. In Figure 5, the recommendation model of the supervised Auto Encoder is built. The model is divided into two steps: feature extraction and reconstruction of the input. To introduce structured data into the model and improve the quality of feature extraction, when objects or users have the same structural attributes, they should have more similar features. For example, multiple objects are action movies. A classification operation is an operation that can extract similarities in data. A new recommendation framework is proposed: a supervised Auto Encoder recommendation model. The structured data of objects or users improves the recommendation effect.

The recommendation model of the SAE is shown in Equation (4):


$$\underset{w}{m\,i\,n}F_{R}\,\left(r,w_{e},w_{r},b_{e},b_{r}\right)+\alpha \,\sum F_{c}\,\left(r,w_{e},w_{c},b_{e},b_{c}\right)$$

(4) $$+\frac{\beta }{2}\,\left({∥w_{e}∥}_{F}^{2}+{∥w_{r}∥}_{F}^{2}+{∥w_{c}∥}_{F}^{2}\right)$$

In Equation (4), *w_*e*_, w_*b*_*, represent the feature extraction stage parameters. *w_*r*_, b_*r*_* represent the parameters of the refactoring phase. *w_*c*_, b_*c*_* define the parameters of the classification stage. *F*_*R*_() represents the loss function in the reconstruction stage. *F*_*C*_() is the loss function in the classification stage. α, β are regularization factors, which are used to control the weights of the items in Equation (4).


(5) $$F_{R}\,\left(r,w_{e},w_{r},b_{e},b_{r}\right)=\sum_{r\in R}{∥r-h\,\left(r,w_{e},w_{r},b_{e},b_{r}\right)∥}_{0}^{2}$$

Equation (5) indicates that only the influence of the observable score is considered. The green part in Figure 5. *h*() represents the entire feature extraction and neural reconstruction network. *h*() is a stacked auto-encoder model accumulated by multiple layers of auto-encoders.


$$F_{C}\,\left(r,w_{e},w_{e},b_{e},b_{c}\right)$$

(6) $$=\left\{ \begin{array}{c} {\left(\text{sigmoid}\left(w_{c}\cdot h_{E}\left(r,w_{e},b_{e}\right)+b_{c}\right)\right)}^{y}\times \\ {\left(1-\text{sigmoid}\,\left(w_{c}\cdot h_{E}\,\left(r,w_{e},b_{e}\right)+b_{c}\right)\right)}^{\left(1-y\right)}\\ \sum_{j=1}^{k}1\left\{y^{\left(i\right)}=j\right\}log\left(\text{softmax}\left(w_{c}\cdot h_{E}\left(r,w_{e},b_{e}\right)+b_{c}\right)\right) \end{array}\right.$$

In Equation (6), *h*() represents the entire feature extraction stage, and the feature extraction stage is composed of multiple accumulated automatic encoders. *F*_*C*_() depends on the class of structured features (binary or multi-class). *F*_*C*_() can help the feature extraction stage to extract relevant information based on its structured features.

In order to overcome the data sparsity problem in recommender systems, regularity constraints based on the Huber function are introduced into the SNR model. The definition of the Huber function is shown in Equation (7):


(7) $$\text{H}\,\left(t\right)=\left\{ \begin{array}{c} t^{2}\,\left|t\right|\leq \mu \\ 2\,\mu \,\left|t\right|-\mu^{2}\,t>\mu \end{array}\right.$$

The objective function of the recommendation model SNR of the PBL online learning system is shown in Equation (8):


$$J=F_{R}\,\left(r,w_{e},w_{r},b_{e},b_{r}\right)+\alpha \,\sum F_{c}\,\left(r,w_{e},w_{c},b_{e},b_{c}\right)$$

(8) $$+\frac{\beta }{2}\,\left(\text{H}\,\left(w_{e}\right)+\text{H}\,\left(w_{r}\right)+\text{H}\,\left(w_{c}\right)\right)$$

H() is the Huber function, and *r* represents the model’s input, that is, the rating vector.

The sparse autoencoder is divided into input layer, hidden layer, and output layer. In the encoder, the sparse autoencoder takes the input vector and encodes it into the hidden layer, thus reducing the dimension of the input layer. The dimension-reduced data is restored to the dimension of the original data by the decoder. Compared with the previous common artificial neural network structure, the sparse autoencoder sets the overall number of layers to 3, and assumes that the vector obtained by its output layer is the same as the input vector of the input layer. In order to ensure the sparsity of the sparse autoencoder, when the dimension of the input vector is high or the number of neurons in the hidden layer is large, a sparsity limit is added to the neurons in the hidden layer during the training process. exact structure. Based on the understanding of the online learning system platform and the analysis of the current application of the online learning platform, a personalized online learning system based on genetic algorithm is designed to provide learners with personalized learning. In the design of the system, the system uses the basic principle of genetic algorithm to find an optimal solution, plans the online learning process, efficiently uses the online learning time, and provides students with personalized learning to improve students’ autonomous learning ability in the network environment. In the online learning system, the individualized feature extraction of learners is the core and key to realize individualized learning. The learning interest of users can be obtained by analyzing the learning pages browsed by learners and extracting the text features of the pages. At present, there are many methods for extracting text features, such as mutual information between words and categories, word entropy between words and categories, and cross entropy. It is not difficult to find that these traditional feature extraction formulas differ only in the choice of keywords. Most of its improvements are based on the frequency of words and the position of words in the document to determine the weight of keywords, which cannot meet the needs of providing personalized information services for different users. In addition, they all require many samples for feature extraction. It is difficult to obtain personalized features under the condition of small samples, and in the online learning system, due to the variability of each user’s information needs, there may be few training samples used for feature extraction. The Genetic Algorithm is used for Feature Extraction Genetic Algorithm is an optimization search method that combines efficiency and effect. From a microscopic point of view, a genetic algorithm is a random algorithm. From a macroscopic point of view, it also has a certain directionality. It starts from a group of points, goes through the steps of selection, hybridization, mutation, etc., and evolves from generation to generation to obtain a satisfactory solution. In the personalized online learning system based on genetic algorithm, the confidence feature is extracted through the genetic algorithm, the personalized information of the learner is obtained, and the learning model of the learner is established.

The recommendation accuracy prediction evaluation criteria of the PBL online learning system are introduced. When measuring the accuracy of score prediction, there are generally two indicators: Root Mean Square Error (RMSE) and Mean Absolute Error (MAE). For user *i* and object *j*, assuming that *r*_*ij*_ is the actual score of user *i* to object j, ${\hat{r}}_{i\,j}$ represents the predicted score of the algorithm, and *T* represents the entire test set, then RMSE and MAE are shown in Equations (9) and (10):


(9) $$\text{RMSE}=\sqrt{\frac{\sum_{i,j\in T}{\left(r_{i}-{\hat{r}}_{i\,j}\right)}^{2}}{\left|T\right|}}$$

(10) $$\text{MAE}=\frac{\sum_{i,j\in T}\left|r_{i\,j}-{\hat{r}}_{i\,j}\right|}{\left|T\right|}$$

### Experiment Preparation

#### Questionnaire Design

The questionnaire design adopted the six-dimensional “entrepreneurial psychological characteristics” (Chen et al., 2020; Feng and Chen, 2020), which was set up from the six dimensions of innovation, control, endurance, risk-taking, self-confidence, and achievement. In the questionnaire, each size contains 3–6 questions, including 1–4 reverse scoring questions, and a total of 25 questions centering on experience learning, cognitive learning, practical learning, entrepreneurial intention, and active personality design.

#### Problem-Based Learning Deep Teaching Practice Design

This method selects two classes of the third-year social security law major in College B that are about to face the problem of entrepreneurship as the research objects, with a total of 86 students. All subjects are divided into experimental and control classes, with 43 students in each category. The practical and management classes received the same test. The teaching item settings are shown in Table 1 (Deng et al., 2021; Liu et al., 2021).

**TABLE 1:** PBL deep learning entrepreneurship education program design.

Week times	Course content	Achievement display
1	Innovative thinking: how to create higher returns with low-cost investment?	Intra group discussion and inter group display.
2	Entrepreneurial opportunities: project thinking combined with social status.	Market Research and analysis, intra group discussion, inter group display.
3	Entrepreneurial project: project planning case writing.	Market Research and analysis, intra group discussion, inter group display.
4	Project design: Taking organizational behavior as an example.	Intra group discussion and inter group display.
5	Business model: case study.	Intra group discussion.
6	Project promotion: publicity and distribution scheme design.	Intra group discussion and inter group display.
7	Team building: financing scheme writing.	Market Research and analysis, intra group discussion, inter group display.
8	Project practice: campus report display.	Campus report display.

## Experimental Design and Performance Evaluation

### Experimental Environment

All experiments use the Python programming language of version 3.6.5. The operating system is Red Hat 4.8.5–28. The CPU is a 16 cores Intel Xeon ^®^ CPU (2.10 GHz). The graphics processor is Tesla P100 PCIe 16 GB. The memory capacity is 64 GB. The main Python packages used are: scikit-learn in version 0.21.3, scipy in version 1.1.0, pandas in version 0.23.0, and TensorFlow in version 1.12.3.

### Description of the Primary Objects of the Questionnaire Survey

The method selected 287 social security law juniors and seniors in College B who are about to face employment and entrepreneurship, and released an electronic version of the questionnaire. In all distributed electronic questionnaire surveys, a total of 263 questionnaires are recovered, with a recovery rate of 91.6%; 259 valid questionnaires are obtained, with a questionnaire response rate of 98.4%. Of the valid survey respondents, 137 women accounted for 53% and 122 men accounted for 47%. For the reliability of the questions in the questionnaire, the method uses a scale reliability test. When Cronbach’s α < 0.7, the question is deleted; when Cronbach’s α is between 0.7 and 0.8, the question has good reliability; when Cronbach’s α > 0.8, the question has high reliability. Meanwhile, this method uses Kaiser-Meyer-Olkin (KMO) value and Bartlett test for data analysis. When the KMO value is greater than 0.7, the Bartlett test value is significant and can be analyzed.

### Reliability Test of Entrepreneurship Psychology Scale and Analysis of Questionnaire Results

**FIGURE 6:**
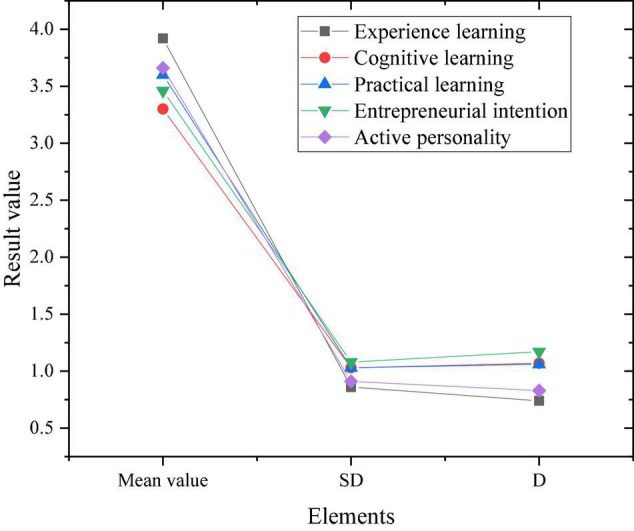
Variable descriptive results.

The mean value, standard deviation (SD) and deviation (D) of the independent variables in the research subject are calculated and analyzed, and the result shown in Figure 6 is obtained.

**FIGURE 7:**
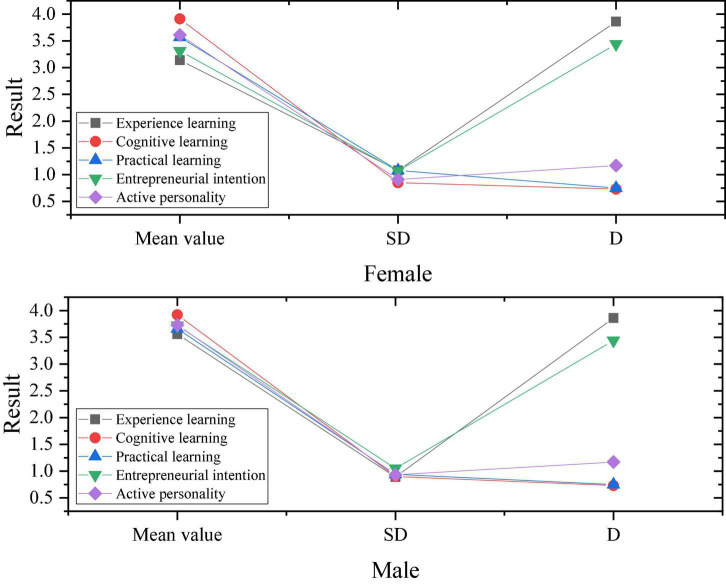
Descriptive results of gender impact on variables.

Figure 6 shows that the mean value of empirical learning in entrepreneurial learning is close to 4, while the SD and D are low, indicating that the students of the social security law major as the research sample attach the highest importance to empirical learning in entrepreneurial learning. The average value of practical learning and active personality is between 3.5 and 4. The SD is also small, indicating that students pay more attention to these aspects. The average value of cognitive learning and entrepreneurial intention is between 3 and 3.5. Students pay less attention to cognitive knowledge and entrepreneurial intention in entrepreneurial learning. For the effect of gender on variables, the results are shown in Figure 7.

**FIGURE 8:**
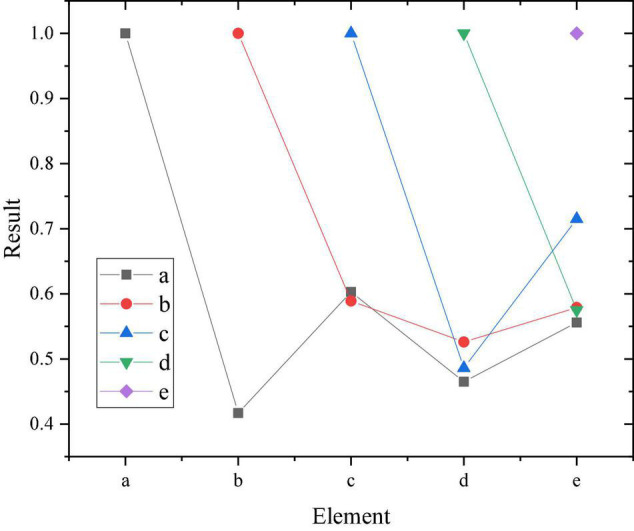
Variable correlation results.

Figure 7 shows that the D of gender as a variable in the experience learning in entrepreneurial learning and entrepreneurial intention in entrepreneurial learning is higher. This result shows that different genders have different influences on the entrepreneurial experience learning and entrepreneurial intentions of college students. In the empirical learning of entrepreneurial learning, the average value of males is higher than that of females, indicating that males pay more attention to empirical learning in entrepreneurial learning than females. The entrepreneurial intentions in the results of the questionnaire also showed obvious differences, indicating that males tend to have stronger entrepreneurial intentions. In order to facilitate the analysis of the correlation between variables, define the experience learning in entrepreneurial learning as a, cognitive learning as b, practical learning as c, entrepreneurial intention as d, and active personality as e. The correlation results are shown in Figure 8.

**FIGURE 9:**
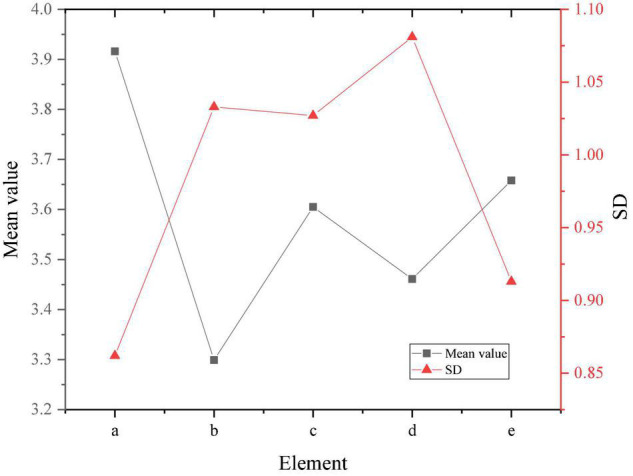
Comparison of variable mean and *SD*.

The obtained mean and SD results is shown in Figure 9.

Figures 8, 9 show that the empirical learning, cognitive learning and practical learning of entrepreneurial learning are all positively correlated with entrepreneurial intentions, and the significance is < 0.01. Active personality and entrepreneurial intentions also show a significant positive correlation, with a significance of < 0.01. Meanwhile, entrepreneurial learning and active personality present a positive correlation, with a significance of < 0.01.

### The Mesomeric Effect Test of Active Personality Between Entrepreneurial Learning and Entrepreneurial Intention

#### Entrepreneurship Learning and Entrepreneurial Intention Test

**FIGURE 10:**
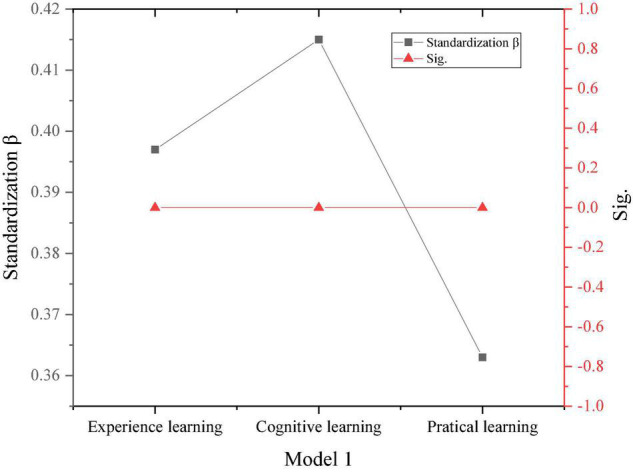
Test of the impact of entrepreneurial learning on entrepreneurial intention.

The proposed method defines the independent variable as entrepreneurial learning, and the dependent variable as entrepreneurial intention, and verifies the influence of entrepreneurial learning on entrepreneurial intention. The results are shown in Figure 10.

**FIGURE 11:**
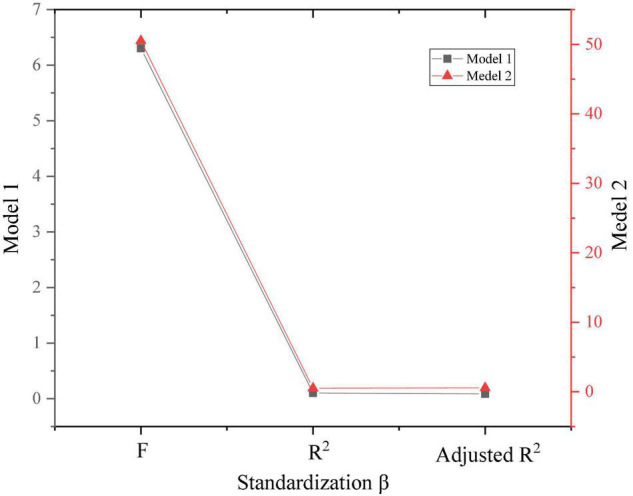
*F*-test.

Additionally, according to Figure 10, the result of the F test is shown in Figure 11.

Figures 10, 11 show that the F of the two models is 6.3 and 50.4, respectively, and *P* < 0.001. After the F test, it is proved that there is a linear relationship between entrepreneurial learning and entrepreneurial intention. After examining the three contents of experience learning, cognitive learning, and practical learning in entrepreneurial learning explains 56% of the sudden changes in entrepreneurial intention, which shows that entrepreneurial learning has played a decisive role in the generation and change of entrepreneurial purpose. Meanwhile, in entrepreneurial learning, the regression coefficient of experience learning is 0.341, the regression coefficient of cognitive learning is 0.418, the regression coefficient of practical learning is 0.359, and *P* < 0.001 for three items. This result shows that the three dimensions of entrepreneurial learning significantly affect entrepreneurial intentions. Therefore, the proposed method assumes that T1, T1a, T1b, and T1c have been verified and the content holds.

#### The Mesomeric Effect of Active Personality Between Entrepreneurial Learning and Entrepreneurial Intention

**FIGURE 12:**
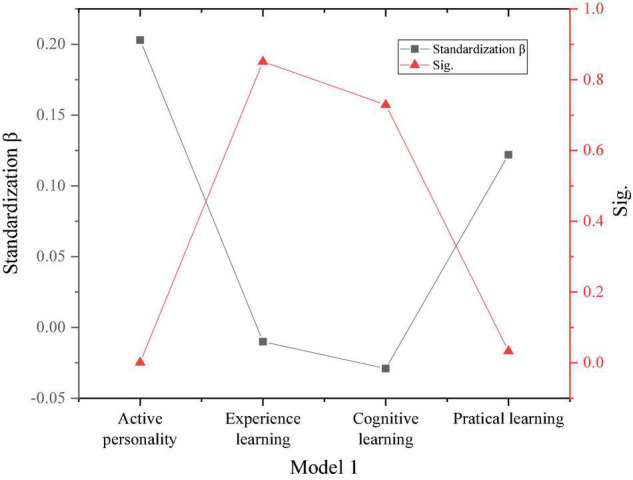
The mediation test of active personality.

The proposed method defines the dependent variable as entrepreneurial intention. The test of the product of the three dimensions of entrepreneurial learning and active personality verifies the mediating role of dynamic character between entrepreneurial education and entrepreneurial intention. The results are shown in Figure 12.

**FIGURE 13:**
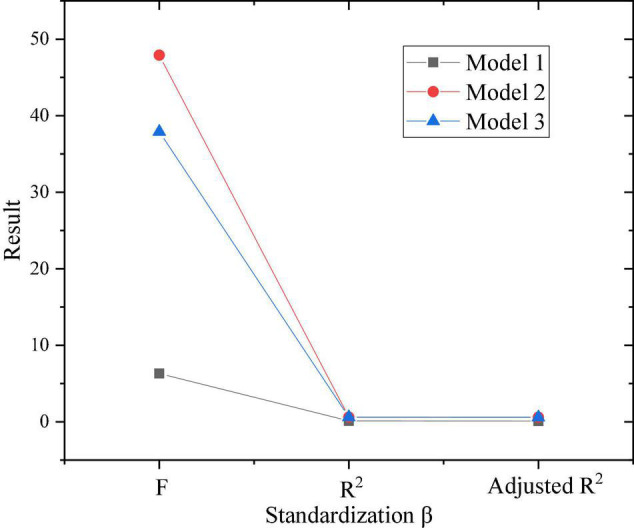
*F*-test results.

Additionally, according to Figure 12, the result of the *F* test is shown in Figure 13.

Figures 12, 13 show that the influence of active personality on experiential learning and cognitive learning is 0.8 and 0.7, and both results are greater than 0.5. Therefore, the mediating role of active personality between experiential learning and cognitive learning and entrepreneurial intention is not obvious. The influence of active personality in practical learning is 0.03, which is less than 0.5. Thus, the active personality can mediate between practical learning and entrepreneurial intentions. The research hypotheses T1 and T1c have been verified, and T1s and T1b have not been verified. Table 2 is the hypothetical regression analysis results.

**TABLE 2:** Hypothetical regression analysis results.

	Variable β	Model	
	Gender	Standardization β	Sig.
Control variable	Grade	0.010*	0.811
	Professional	0.012*	0.746
	Household registration	0.148**	0.000
	Friends and relatives start a business	0.108**	0.004
	Entrepreneurial experience	−0.024*	0.526
	Entrepreneurial resilience	−0.103*	0.012
Standard variable	Entrepreneurial power	0.123**	0.007
	Active personality	0.224***	0.001
	F	6.304***	
	*R* ^2^	0.587	

***p < 0.001, **p < 0.01, *p < 0.05.

In Table 2, the F value of the model is 6.304, and the *P*-value is less than 0.001. Through the *F* test, it shows that there is a significant linear relationship between entrepreneurial learning and entrepreneurial intention. The significance levels of the control variables household registration and entrepreneurial experience in the model are 0.001 and 0.000, respectively, indicating that household registration, entrepreneurial experience, and entrepreneurial intention are significantly correlated. In addition, entrepreneurial resilience, entrepreneurial strength, and optimism can effectively explain 57.5% of the variance of entrepreneurial intention, indicating that entrepreneurial resilience plays a decisive role in entrepreneurial intention. Entrepreneurial resilience (β = −0.103, *P* < 0.01), entrepreneurial strength (β = 0.123, *P* < 0.001), and proactive personality (β = 0.224, *P* < 0.001) significantly influenced entrepreneurial intention. Therefore, T2, T2a, T2b, T2c pass the test.

The deep implicit relationship in the variables is found through further analysis of the survey results in the questionnaire. New hidden variables are found and added to the model through this implicit relationship.

### Problem-Based Learning Method Teaching Experiment and Result Analysis

**FIGURE 14:**
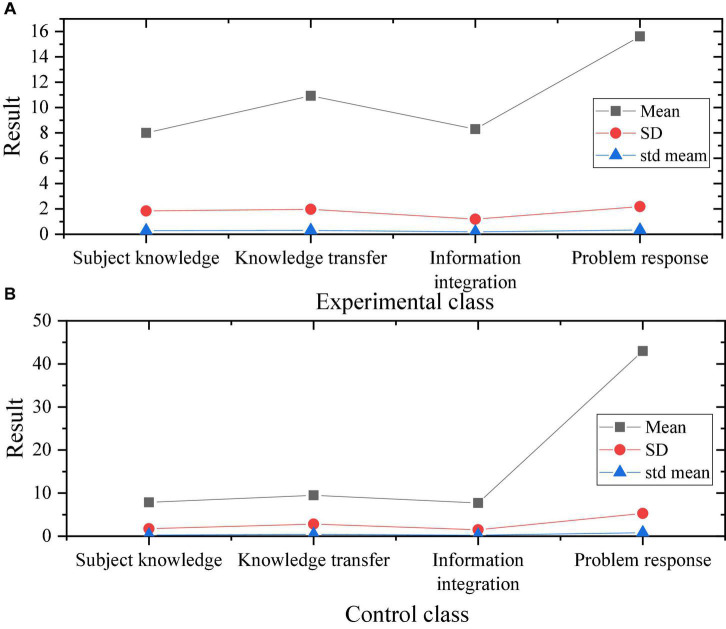
Test of results of experimental class and control class. **(A)** Sample test of experimental class; **(B)** sample test of control class.

An 8-week entrepreneurial education is carried out for the experimental class using the research-designed program, compared with the traditional curriculum program, and tested and analyzed. After the course is over, the students in the experimental class are evaluated from four aspects: knowledge mastery, knowledge transfer, knowledge integration, and problem response. The test results obtained are shown in Figure 14.

**FIGURE 15:**
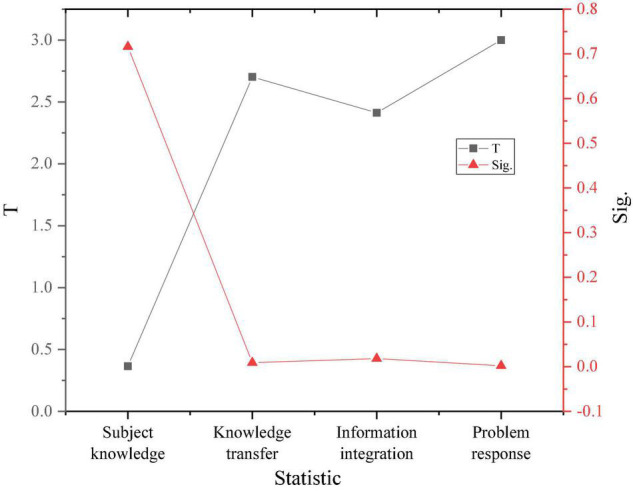
Test of *t*-value and Sig. result of experimental class and control class.

In Figure 14, the t and Sig. of the two class scores are obtained, and the test is shown in Figure 15.

Figures 14, 15 reveal that there is no significant difference in knowledge mastery between the experimental class and the control class. This result shows that after passing entrepreneurship education, students in both classes can master the basic knowledge of entrepreneurship education. On the transfer of knowledge: the Sig value of the students in the experimental class is 0.08, which is less than 0.05. The results show that: PBL deep learning curriculum program can improve students’ knowledge transferability and better apply and integrate subject knowledge into practice. For the integration of knowledge, the Sig. of the experimental class and the control class are both lower than 0.05, but there are obvious differences. The results show that: PBL courses have improved and strengthened students’ information integration ability. Meanwhile, the problem response of the two groups of classes is less than 0.05, and the mean value of the experimental class is greater than that of the control class. The results show that: the PBL course program can improve students’ teamwork and problem response ability. The students trained in the PBL course have shown a significant improvement in their entrepreneurial ability, can plan and write, can better coordinate the work between groups, and their comprehensive literacy has been significantly improved.

### Implications and Recommendations From the Research

Entrepreneurial learning has a significant positive effect on entrepreneurial intention. Specifically, the influence of each dimension of entrepreneurial education on college students’ entrepreneurial intention is cognitive learning, experiential learning, and practical learning from high to low. This is consistent with the theory of planned behavior. When college students start a business, they need to make a series of preliminary preparations, including various resources such as funds, venues, and policies. Therefore, these influencing factors negatively impact college students’ entrepreneurial intentions. Entrepreneurial learning has a significant positive effect on entrepreneurial resilience. Entrepreneurial resilience has a significant positive effect on college students’ entrepreneurial intention. This is consistent with self-efficacy theory. The results show that entrepreneurial resilience partially mediates the three dimensions of entrepreneurial learning and college students’ entrepreneurial intentions. This suggests that entrepreneurial learning has a direct impact on entrepreneurial intentions. At the same time, entrepreneurial resilience indirectly impacts entrepreneurial intention. A proactive personality can adjust the entrepreneurial elasticity, intensity, and entrepreneurial purpose of college students. Therefore, whether college students have an active character plays a decisive role in generating entrepreneurial intention.

### Problem-Based Learning Online Learning Recommendation System Analysis

**FIGURE 16:**
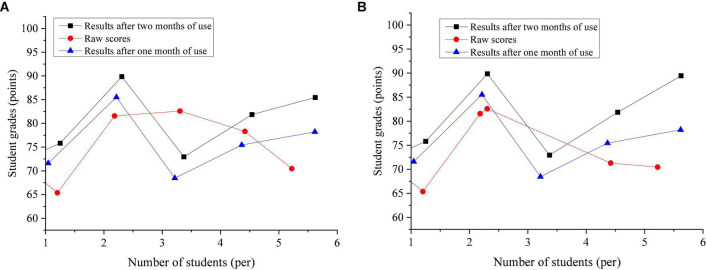
Changes in grades. **(A)** The first test **(B)**. The second test.

Figure 16 shows the actual grades of five students after using the system and the grades when they are not using the system.

The results of changes in student performance are shown in Figure 16. Take Student 1 as an example, his original score is 68. After the 1-month experiment, the score of Student 1 became 70 points, and after the 2-month experiment, the score of Student 1 became 72 points. Overall, the proposed system can improve student performance to a certain extent.

The proposed SNR model is tested offline on two classic recommender system datasets, MovieLens-100K and MovieLens-1M datasets.^1^ Literature (Li et al., 2015) is compared with those in Table 3.

**TABLE 3:** Comparison of the errors of the proposed algorithms in the two databases and the algorithm in the literature (Li et al., 2015).

Contrast algorithm	RMSE		MAE	
	MovieLens-100K	MovieLens-1M	MovieLens-100K	MovieLens-1M
SNR (this study)	0.8854 ± 0.0028	0.8373 ± 0.0012	0.659	0.611
Literature Li et al. (2015)	0.9024 ± 0.0030	0.8407 ± 0.0011	0.680	0.637

In Table 2, the SNR model achieves the best results in the comparative experiments. Compared with the model literature (Li et al., 2015) that also uses structured information to improve the recommendation effect, the SNR model only uses the structured data of the movie category. Still, it performs better than the literature (Li et al., 2015). This shows the superiority of its structured information extraction method. SNR utilizes structured information and Huber function-based priors to improve the recommendation accuracy of the model effectively. Therefore, the collaborative filtering recommendation framework of SNR is very effective.

The contribution of this study to PBL research lies in the use of the method of creating problem situations to improve students’ initiative in knowledge construction; the use of multi-angle evaluation and reflection methods to help students generate higher-order thinking such as critical thinking. This allows learners to flexibly transfer existing knowledge to new situations to creatively solve problems and achieve the high-level goals of deep learning.

When scholars use the Theory of Planned Behavior (TPB) theory to study entrepreneurial activities, they often directly transplant the theory into the field of entrepreneurship. They did not propose new variables according to research characteristics in the field of entrepreneurship. In this way, when interpreting the research results, it is easy to cause unclear effects of variable indications, which is not conducive to the sharing of theories among different scholars. Here, according to the existing research theoretical basis, the corresponding concepts in the entrepreneurship field of the concepts involved in the TPB theory are found. The TPB theory is more suitable for the study of entrepreneurial activities.

## Conclusion

With the advent of the era of information intelligence, today’s society puts forward higher requirements for talent training. It requires students to be able to learn independently, to have the high-order thinking of actively constructing knowledge, analyzing problems, and solving problems. The learning method should be transformed from simple and formal shallow learning to creative and critical deep learning. PBL and deep learning have strong suitability and compatibility, and it is an effective way to realize deep learning. Students majoring in social security law in College B are used as research samples, and the relationship between optimistic personality, entrepreneurial learning and entrepreneurial intention is investigated and analyzed. A special mediating effect between optimistic personality and entrepreneurial intention is verified. Furthermore, a deep learning scheme based on PBL mode is proposed. Through the research, it is concluded that: 1. students who have undergone PBL deep learning can understand the knowledge imparted in entrepreneurship education, and have better integration and output capabilities for the acquired knowledge. Meanwhile, these students can make better coping behaviors when dealing with entrepreneurial problems and changes in the entrepreneurial environment, and achieve the goals of deep learning. 2. The PBL online learning system has proved the effectiveness of the proposed SNR model in the recommendation of the PBL online learning system by comparing different datasets. 3. In the experiential learning of entrepreneurship learning, the average value of men is higher than that of women. It shows that men pay more attention to experiential learning in entrepreneurial learning than women. Entrepreneurial intentions also showed obvious differences, indicating that men tend to have stronger entrepreneurial intentions.

Some deficiencies remain: only one university is selected as a sample at the time of the study. Therefore, there is still a certain bias in the research of this specialty. In future studies, the sample size will be increased and the sample diversity will be further refined. In addition, due to the lack of theoretical research and teaching experience, the teaching experience is relatively lacking, and there are certain defects in the application of the designed method. This deficiency will continue to be studied in the follow-up work.

## Data Availability Statement

The raw data supporting the conclusions of this article will be made available by the authors, without undue reservation.

## Ethics Statement

The studies involving human participants were reviewed and approved by the Shanghai University of Finance and Economics Ethics Committee. The patients/participants provided their written informed consent to participate in this study. Written informed consent was obtained from the individual(s) for the publication of any potentially identifiable images or data included in this article.

## Author Contributions

Both authors listed have made a substantial, direct, and intellectual contribution to the work, and approved it for publication.

## Conflict of Interest

The authors declare that the research was conducted in the absence of any commercial or financial relationships that could be construed as a potential conflict of interest.

## Publisher’s Note

All claims expressed in this article are solely those of the authors and do not necessarily represent those of their affiliated organizations, or those of the publisher, the editors and the reviewers. Any product that may be evaluated in this article, or claim that may be made by its manufacturer, is not guaranteed or endorsed by the publisher.

## Footnotes

1
https://grouplens.org/datasets/moviele.ns
